# Supplementary material for: Subtle left-right asymmetry of gene expression profiles in embryonic and foetal human brains
Source: Sci Rep. 2018 Sep 4;8:12606. doi: 10.1038/s41598-018-29496-2 (PMC6123426; doi:10.1038/s41598-018-29496-2)
Supplement: Supplementary file 1 — Supplementary Information [file 41598_2018_29496_MOESM1_ESM.docx]

Supplementary Results

Subtle left-right asymmetry of gene expression profiles in embryonic and foetal human brains

Carolien G.F. de Kovel, Steven N. Lisgo, Simon E. Fisher, Clyde Francks

Contents

[Midbrain and forebrain, 5-5.5 post conception weeks 1](#_Toc516480736)

[Forebrain laterality at 5-5.5 post conception weeks 2](#_Toc516480737)

[Midbrain laterality at 5-5.5 post conception weeks 3](#_Toc516480738)

[Comparing left-right asymmetry in the midbrain and forebrain age 5-5.5 post-conception weeks 4](#_Toc516480739)

[Brain structures in foetuses aged 7.5-13 post conception weeks 4](#_Toc516480740)

[General observations not considering laterality at 7.5-13 post conception weeks 4](#_Toc516480741)

[Laterality in foetuses aged 7.5-13 post conception weeks 6](#_Toc516480742)

[Permutations swapping sides 8](#_Toc516480743)

[Supplementary Tables 9](#_Toc516480744)

[Supplementary References 9](#_Toc516480745)

[Supplementary Figures 11](#_Toc516480746)

## Midbrain and forebrain, 5-5.5 post conception weeks

*General observations not considering laterality*

The expression profiles for midbrain and forebrain were overall very similar with correlation R=0.97 (i.e. those genes with higher expression in one structure also tended to be high in the other, and vice versa). Yet, in differential expression analysis comparing the two structures bilaterally, 7,262 out of 14,856 genes detected in both structures showed differential expression with FDR < 0.05. MDS analysis also showed a separation between midbrain and forebrain (Supp. Fig. 2). GSEA revealed that the midbrain showed higher expression than the forebrain of genes involved in synaptic signalling, neurotransmitter transport and other GO-terms related to differentiated neural functions (Supp. Table 4). The midbrain showed lower expression than the forebrain of genes in translation/protein-production related GO-terms, such as ‘establishment of protein localization to ER’ and ‘translational initiation’ (Supp. Table 4).

The bilateral expression profiles for our newly sequenced 5-5.5pcw embryos (i.e. where we had separated left from right) and the profiles for the previously sequenced 4.5-9pcw embryos (no left-right dissection) were similar, with correlation of expression levels between the two datasets R=0.84 for forebrain and R=0.80 for midbrain (i.e. within each structure, relative levels of per-gene expression were similar as assessed over all expressed genes; p-values <1E-200). In the 4.5-9pcw dataset, both midbrain and forebrain showed a decrease of bilateral expression of genes involved in the cell cycle with increasing age, and an increase of genes involved in synaptic signalling with age (Supp. Table 3). Changes in gene expression with age were correlated between forebrain and midbrain (R=0.723, p<1E-200)).

### Forebrain laterality at 5-5.5 post conception weeks

Multi-dimensional scaling of gene expression data from the 5-5.5pcw forebrain samples showed the second dimension separating the female samples from the male samples (t-test, p=0.002) (Supp. Fig. 3). The third dimension, though not separating the two sides as such, placed the left sides consistently shifted with respect to the matching right sides (paired t-test, p = 0.0003).

Three individual genes showed significant laterality with FDR < 0.05 in left-right differential expression analysis of this structure: *KCTD12* (FC (fold change)=1.3, FDR=0.03), *SNAI1* (FC=1.3, FDR=0.03) and *GATA2* (FC=1.5, FDR=0.03), all three with higher expression in the right forebrain than the left. Note that we did not perform further correction for multiple testing in relation to all the separate analyses in this study, and therefore these individual gene findings in the embryonic forebrain remain tentative. In the dataset of forebrains aged 4.5-9pcw which were not separated into left and right, *SNAI1* expression suggestively decreased with age (p(nom)=0.003), while *GATA2* (p(nom)=0.47) and *KCTD12* (p(nom)=0.21) showed no age-related change. *GATA2* is a transcription factor, but its target gene-set showed no differential left-right expression in the forebrain at 5-5.5pcw, (target set V$GATA2_01 with 104 target genes, Normalised Enrichment Score (NES)=-1.0 left, p(nom)=0.003, FWER=0.999). *SNAI1* is also a transcription factor but the MsigDB v5.2 database contained no target gene sets defined for this gene.

Forebrains of 5-5.5pcw embryos clearly showed lateralisation at the level of GO gene-sets. In GSEA, 1157 gene-sets showed left-right differences with FDR < 0.25, whereas when differential expression t-values were permuted among genes, the average number of GO-terms with FDR< 0.25 in each permutation was only 1.1. Notably, GO-terms associated with skeletal structure and with blood vessel formation showed higher expression on the right, amongst others. On the left, only two GO-terms showed significantly higher expression, which were 'cerebral cortex neuron differentiation' and 'establishment of mitochondrion localisation' (Figure 2, Supp. Table 1). Enrichment analysis for transcription factor targets showed higher expression on the left of targets of *REST* (NRSF) and on the right of *FOXJ1* targets (Supp. Table 2)*.* *REST* encodes a transcriptional repressor of neuronal genes in non-neuronal tissues ^1,2^. *REST* itself showed some degree of lateralisation itself as well (FC=1.17 to the right, p(nom)=0.0003; the laterality of *REST* is expected to be opposite to its target gene-set because this TF is a negative regulator of expression), while *FOXJ1* itself showed no obvious lateralisation (FC=1.10 to the right, p(nom)= 0.374).

Comparison of the laterality results from the 5-5.5pcw embryos with the non-left-right-separated forebrains of 4.5-9pcw showed that none of the most lateralised GO-sets had strong relations with age. Across all genes detected in both datasets (N=12,556), the correlation between left-right and age effects was very low (R=-0.04, p=1.9E-05; Figure 1, Figure 2). *REST* showed no clear relation with age (p(nom)=0.14), while *FOXJ1* suggestively increased with age (p(nom)=0.04).

### Midbrain laterality at 5-5.5 post conception weeks

In MDS analysis of the newly generated data for 5-5.5pcw midbrain samples (i.e. with left-right separation), the first dimension separated the sexes (t-test, p=7.2E-05; Supp. Fig. 3). None of the dimensions separated the sides.

One individual gene was expressed significantly more highly in the left midbrain relative to the right (FC=1.3, FDR = 0.03), which was *SOX1,* although again we did not correct further for all of the different analyses in this study. Though *SOX1* is a transcription factor, MSigDB provided no target gene-set. Midbrains of 5-5pcw clearly showed lateralisation in terms of GO gene-sets. In the GSEA analysis, 573 gene-sets showed left-right differences with FDR < 0.25, but when laterality t-values were permuted among genes, the average number of GO-terms in each permutation with FDR< 0.25 was only 2.6. In the left midbrain, the terms ‘cell fate specification’ and ‘cell fate commitment’ showed higher expression, consistent with the known functions of *SOX1* in cell fate determination ^3,4^*.* However, the most significant GO-term was ‘nephron tubule formation’. No GO-terms were significantly more highly expressed in the right midbrain than the left (Supp. Table 1). Enrichment analysis for transcription factor targets showed higher expression on the left of targets of *SP1* (Supp. Table 2)*.* The gene *SP1*, though highly expressed, showed no asymmetry itself (FC=1.03 right, p(nom)=0.44).

In the data of midbrains ranging from 4.5-9pcw (i.e. not separated into left and right), the genes increasing with age were enriched for GO-terms for synaptic signalling, neurotransmitter transport and other terms related to differentiated neurons, while decreasing with age were GO-terms for DNA replication and cell cycle (Supp. Table 3). Across all genes (N=13,080) detected in the 5-5.5pcw embryos with left-right separation and the 4.5-9pcw embryos with no left-right separation, the correlation between left-right and age effects was R=0.262 (p< 1E-200; Figure 1. In other words, the right was slightly ahead of the left in terms of the age-dependent changes which both sides followed (Figure 2).

### Comparing left-right asymmetry in the midbrain and forebrain age 5-5.5 post-conception weeks

Left-right differences between forebrain and midbrain were not correlated over all genes (correlation of left-right differential expression t-values between the two structures, R=0.0001; p=0.22). In contrast to the midbrain, *SOX1* showed no clear lateralisation in the forebrain (FC=1.10 left, p(nom)=0.14), although it was well expressed bilaterally in forebrain (> 7 log2(cpm)). *GATA2, KCTD12* and *SNAI1* which showed laterality in the forebrain, all had moderate to high expression (> 4 log2(cpm)) in midbrain, but showed no strong asymmetry of expression in midbrain (FC=1.14 left; FC=1.01 right; FC=1.13 left, resp. with all p(nom)> 0.18).

## Brain structures in foetuses aged 7.5-13 post conception weeks

In foetuses aged 7.5-13pcw, left-right data were available for four substructures of the forebrain: cerebral minus temporal cortex, temporal cortex, basal ganglia, and diencephalon. An additional structure was the choroid plexus of the lateral ventricles ^5^.

### General observations not considering laterality at 7.5-13 post conception weeks

When considering only those genes detected in all five structures (N=13,441), the choroid plexus of the lateral ventricles showed the least similarity with the other structures: Correlations in overall gene expression of the choroid plexus of the lateral ventricles with the other structures ranged from R=0.62 to R=0.67, whereas correlations between all other structures had R-values exceeding 0.90. In MDS analysis (Supp. Fig. 4) and heat-plots (not shown), choroid plexus was also distinct from the other structures, such that the first MDS dimension separated the choroid plexus from the other structures. The remaining structures were ordered along the second dimension, with cerebral minus temporal cortex on one end, and diencephalon on the other (Supp. Fig. 4).

When contrasting the choroid plexus with the basal ganglia (the latter to represent the other structures whose gene expression profiles were similar), choroid plexus showed lower expression of genes in the GO-terms ‘homophilic cell adhesion via plasma membrane adhesion molecules’, ‘synaptic signalling’, dendrite morphogenesis’ and other GO-terms related to neuronal development or function. The choroid plexus showed higher expression of cilium-related GO-terms such as ’axoneme assembly’, ‘cilium movement’, or ‘cilium organization', as well as other terms, including some involved in cellular respiration (Supp. Table 5). Furthermore, in the choroid plexus, 1,015 genes were detected that were not detected in the other structures. These genes were enriched relative to all known genes for the GO terms ‘interferon-gamma-mediated signalling pathway’, ‘positive regulation of inflammatory response’, ‘toll-like receptor signalling pathway’, ‘leukocyte chemotaxis’, ‘inflammatory response’ and others (Supp. Table 5).

Across all five structures, the expression levels of more than half of all genes (7,577 out of 13,441 which were expressed in all structures) were significantly associated with foetal age at FDR < 0.05. These were roughly equally divided into genes which increased or decreased in expression with increasing age. The genes which increased in expression with age were enriched for GO-terms ‘synaptic signalling’ and other sets related to differentiated neuronal functions, while the genes which decreased in expression with age were enriched for GO-terms related to cell proliferation (cell cycle and division), with ‘nuclear chromosome segregation’ as the most significant term (Supp. Table 3). When analysing the effects of age within each structure separately, the cerebral minus temporal cortex and the diencephalon again showed the overall pattern that genes increasing in expression with age were enriched for synaptic signalling functions, while genes decreasing with age were enriched for mitosis-related functions (Supp. Table 3). However, in the temporal cortex, only a small number of GO-terms were significantly associated with age at FWER < 0.05 (Supp. Table 3), including ‘collagen fibril organization’ and ‘multicellular organismal macromolecule metabolic process’ which decreased with age, and ‘Regulation of polymerization’ which increased with age (Supp. Table 3). The fact that the age range was relatively limited for the temporal cortex (9-12pcw, see Table 1) probably contributed to the limited number of age-related GO-terms in this structure. In the basal ganglia, increasing in expression with age were genes in a variety of GO-terms, with the most significant enrichment for the term 'response to type I interferon', while genes decreasing with age were found in GO-terms for RNA and DNA processing (Supp. Table 3). In the choroid plexus, genes increasing with age were enriched for immunological functions such as 'adaptive immune response' and 'response to interferon gamma' (Supp. Table 3), while decreasing in expression with age were genes involved in ciliary biology such as 'cilium organisation' and 'cilium morphogenesis' (Supp. Table 3).

Across all five structures combined, 26 X-chromosomal genes were differentially expressed between males and females, with only *ANOS1 (KAL1)* higher in males, all others higher in females. Two genes from the pseudo-autosomal region on X and Y, and 13 Y-chromosomal genes were differentially expressed between the sexes, all more highly in males. Eighty-three autosomal genes were differentially expressed between males and females as assessed across all structures, of which twenty-six were higher in males. GO enrichment analysis relative to all known genes showed no enrichment for any process or function, for the genes whose expression differed between the sexes (Supp. Table 6).

### Laterality in foetuses aged 7.5-13 post conception weeks

Table 1 indicates the numbers of foetuses for which we had data for laterality analysis after quality control, which ranged from five foetuses for the temporal cortex to thirteen for the diencephalon (Table I). MDS analysis within each structure separately showed that, among the top six dimensions, there were usually dimensions which correlated with either age, sex, or both (Supp. Fig. 7), but none which correlated with side.

However, four of the structures showed highly significant negative correlations between the effects of age and side on gene expression, such that genes increasing in expression with age had a tendency to be more strongly expressed on the left in the temporal cortex R=-0.187, p=2E-82, basal ganglia R=-0.221, p=2E-162, diencephalon R=-0.240, p=1E-174, and choroid plexus R=-0.361, p<1E-200 (Figure 1. In other words, for these four structures, the left was slightly ahead of the right in terms of the age-dependent changes which both sides followed (Figure 3). In contrast, for the cerebral minus temporal cortex, a positive correlation was found between the overall effects of age and side on gene expression, R=0.174, p=6E-88, (Figure 1), i.e. genes increasing with age had a tendency to be more strongly expressed on the right, such that the right was slightly ahead of the left in terms of the age-dependent changes which both sides followed. Interestingly, this is the reversed asynchrony compared to the temporal cortex (Figure 3).

No individual genes showed significant differential expression between the left and right sides in any of the five foetal brain structures analysed, after FDR correction. However, all five structures showed highly significant laterality of gene expression at the level of functional gene sets (Supp. Table 1).

In the cerebral minus temporal cortex, there was enrichment on the right for GO-terms related to protein translation, while on the left various terms related to the development of blood vessels, and to extracellular structure organisation, showed higher expression, in addition to other terms (Supp. Table 1). In total 1210 GO-terms showed laterality FDR < 0.25, versus 3.1 on average in the randomised permutations. Targets of the transcription factors *GATA1* (two motifs), TEAD1, FOXD1, EVI1 (*MECOM*) and SRF showed higher expression on the left (Supp. Table 2). However, the mRNAs for *GATA1* and *FOXD1* themselves were not detected, while *TEAD1* (FC=1.06 left, p(nom)=0.67), *SRF* (FC=1.03 right, p(nom)=0.63) and *MECOM* (FC=1.33, p(nom)=0.20) showed no obvious lateralisation of expression themselves.

For the temporal cortex, there was higher left-sided expression of synaptic signalling and related terms, as well as cell-cell adhesion. On the right, the most significantly higher expression was found for GO-terms involved in chromosome segregation and other terms related to the cell cycle (Supp. Table 1). In this analysis, 1649 out of 3681 GO-terms had FDR < 0.25, against an average of only 0.4 in the permutations. On the left the targets of the TF NRSF *(REST*) showed higher expression than on the right (*s*ee the results on the forebrain at 5-5.5pcw for information regarding this TF). Also, targets of NF1 (V$MYOGNF1_01) showed higher expression on the left, although *NF1* expression itself was close to symmetrical (FC=1.05 left, p(nom)=0.62). Targets of the E2F family of TFs were expressed more highly on the right (Supp. Table 2). As TFs from the E2F family play crucial roles in the control of the cell cycle ^6,7^, this is consistent with the cell cycle GO-terms being higher on the right (Supp. Table 2). Neither *REST* nor the TFs in the E2F family showed strong lateralisation themselves (*REST,* FC=1.13 right, p(nom)=0.28; *E2F1*, FC=2.42 right, p(nom)=0.08; *TFDP1*, FC=1.11 right, p(nom)=0.42; *TFDP2*, FC=1.4 right, p(nom)=0.10) .

The basal ganglia showed higher expression on the right of GO-terms related to the cell cycle and RNA-processing. The most significantly highly expressed terms on the left included ‘regulation of catecholamine secretion’ and its child term ‘regulation of dopamine secretion’ (Supp. Table 1). In addition, terms related to cellular respiration were expressed more highly on the left, as well as 'oxidative phosphorylation' (Supp. Table 1). In this structure, 1116 GO-terms had FDR < 0.25, against an average of only 1.8 in the permutations. Targets of the transcription factors TLX2, YY1, TFDP1, STAT6 and IRF1 were expressed more strongly on the right, while no target sets showed stronger expression on the left (Supp. Table 2). The mRNAs of *YY1*, *TFDP1*, *STAT6* and *IRF1* themselves did not show obvious asymmetry, (*YY1*: FC=1.03 right, p(nom)=0.36; *TFDP1*: FC=1.02 right, p(nom)=0.24; *STAT6:* FC=1.02 right, p=0.89; *IRF1*: FC=1.04 right, p(nom)=0.76), and *TLX2* was not detected.

In the diencephalon, more highly expressed on the right were a number of cell cycle GO-terms including 'DNA replication' (Supp. Table 1). The GO-terms 'metanephros morphogenesis' and 'oligodendrocyte differentiation' were also more highly expressed on the right (Supp. Table 1). No GO-terms were significantly expressed more highly in the left diencephalon. In total, 919 gene-sets were different between left and right with FDR < 0.25, against only 0.3 on average in the permutations. More highly expressed on the left were the targets of transcription factors AR (androgen receptor, X-chromosomal) and NFIL3 (Supp. Table 2). Higher on the right were target sets for a number of transcription factors in the E2F family, consistent with the cell cycle GO-terms (Supp. Table 2). None of AR, NFIL3 or the E2F family members showed strong lateralisation themselves (*AR:* FC=1.25 right, p(nom)=0.47*; NFIL3:* FC=1.06 left, p(nom)=0.59; *TFDP1*: FC=1.01 right, p(nom)=0.13; *TFDP2*: FC=1.3 right, p(nom)=0.4; *E2F1*: FC=1.51 right, p(nom)=0.35; *E2F4*: FC=1.08 right, p(nom)=0.70).

In the choroid plexus, the right side had higher expression of genes that largely comprised two main functional types (Supp. Table 1): One was related to cilia organisation and movement, while the second was a group of terms related to the cell cycle. Genes showing higher expression on the left side fell into various GO-terms related to the extracellular matrix, but also into some other terms including ‘regulation of rho-protein signal transduction’ and ‘negative regulation of innate immune response’ (Supp. Table 1). 1013 gene-sets had left-right differential expression FDR < 0.25, against only 1.9 on average in the permutations. More highly expressed on the left were also targets of a TF called TFAP2C (Supp. Table 2). The gene *TFAP2C* itself was however not detected in choroid plexus of the lateral ventricles, though it was detected in some other structures including cerebral minus temporal cortex. On the right no TF target sets showed significantly higher expression.

## Permutations swapping sides

These permutations (see methods) showed that on average the true data had the strongest correlation between effects of side on expression and the effects of age on expression (Supp. Fig. 6). The more paired samples were left-right flipped, the weaker the correlation became on average, until more than half of the samples were flipped and the correlation became stronger in the opposite direction. Around the average, however, there was a broad variance, showing that some individual permutations could have even stronger age-side correlations than the true dataset. Though the patterns show that the correlation between effects of side and effects of age is likely to be a real biological phenomenon, the variance suggests that the absolute magnitude of the correlations cannot be accurately assessed in these relatively small sample sizes (Supp. Fig. 6). A similar but more noisy pattern was seen for the number of GO-terms with FDR<0.25 for laterality, arising from these side-flipping permutations (not shown), in which the numbers of significant GO-terms tended to increase the closer a permutation was to the true dataset.

## Supplementary Tables

(Separate excel files)

Supp. Table 1. GO-terms_per_tissue_for_side.xlsx. Per structure, all GO-terms (MSigDB 5.2) enriched with FWER < 0.05 in the left-right differential expression analysis.

Supp. Table 2. TFT_per_tissue_for_side.xlsx Per structure, all transcription factor target (TFT) sets (MSigDB 5.2) enriched with FWER < 0.05 in the left-right differential expression analysis.

Supp. Table 3. GO-terms_per_tissue_for_age.xlsx. Per structure, all GO-terms (MSigDB 5.2) enriched with FWER < 0.05 in the age differential expression analysis.

Supp. Table 4. GO-terms_FB_vs_MB.xlsx. All GO-terms (MSigDB 5.2) enriched with FWER < 0.05 in the differential expression analysis between forebrain and midbrain.

Supp. Table 5. Choroid_plexus_specific.xlsx Genes uniquely detected in choroid plexus (sheet 1) and GO-terms (MSigDB 5.2) enriched or depleted in choroid plexus vs basal ganglia (sheet 2).

Supp. Table 6. Sex_differential_genes.xlsx. Computed over all structures combined, genes with higher expression in one of the sexes with FDR < 0.05.

## Supplementary References

1 Yeo, M. *et al.* Small CTD phosphatases function in silencing neuronal gene expression. *Science* **307**, 596-600, doi:10.1126/science.1100801 (2005).

2 Thiel, G., Lietz, M. & Cramer, M. Biological activity and modular structure of RE-1-silencing transcription factor (REST), a repressor of neuronal genes. *The Journal of biological chemistry* **273**, 26891-26899 (1998).

3 Kan, L. *et al.* Dual Function of Sox1 in Telencephalic Progenitor Cells. *Developmental biology* **310**, 85-98, doi:10.1016/j.ydbio.2007.07.026 (2007).

4 Archer, T. C., Jin, J. & Casey, E. S. Interaction of Sox1, Sox2, Sox3 and Oct4 during primary neurogenesis. *Dev Biol* **350**, 429-440, doi:10.1016/j.ydbio.2010.12.013 (2011).

5 Lindsay, S. J. *et al.* HDBR Expression: A Unique Resource for Global and Individual Gene Expression Studies during Early Human Brain Development. *Front Neuroanat* **10**, 86, doi:10.3389/fnana.2016.00086 (2016).

6 Ertosun, M. G., Hapil, F. Z. & Osman Nidai, O. E2F1 transcription factor and its impact on growth factor and cytokine signaling. *Cytokine Growth Factor Rev* **31**, 17-25, doi:10.1016/j.cytogfr.2016.02.001 (2016).

7 Phillips, A. C., Ernst, M. K., Bates, S., Rice, N. R. & Vousden, K. H. E2F-1 potentiates cell death by blocking antiapoptotic signaling pathways. *Molecular cell* **4**, 771-781 (1999).

## Supplementary Figures


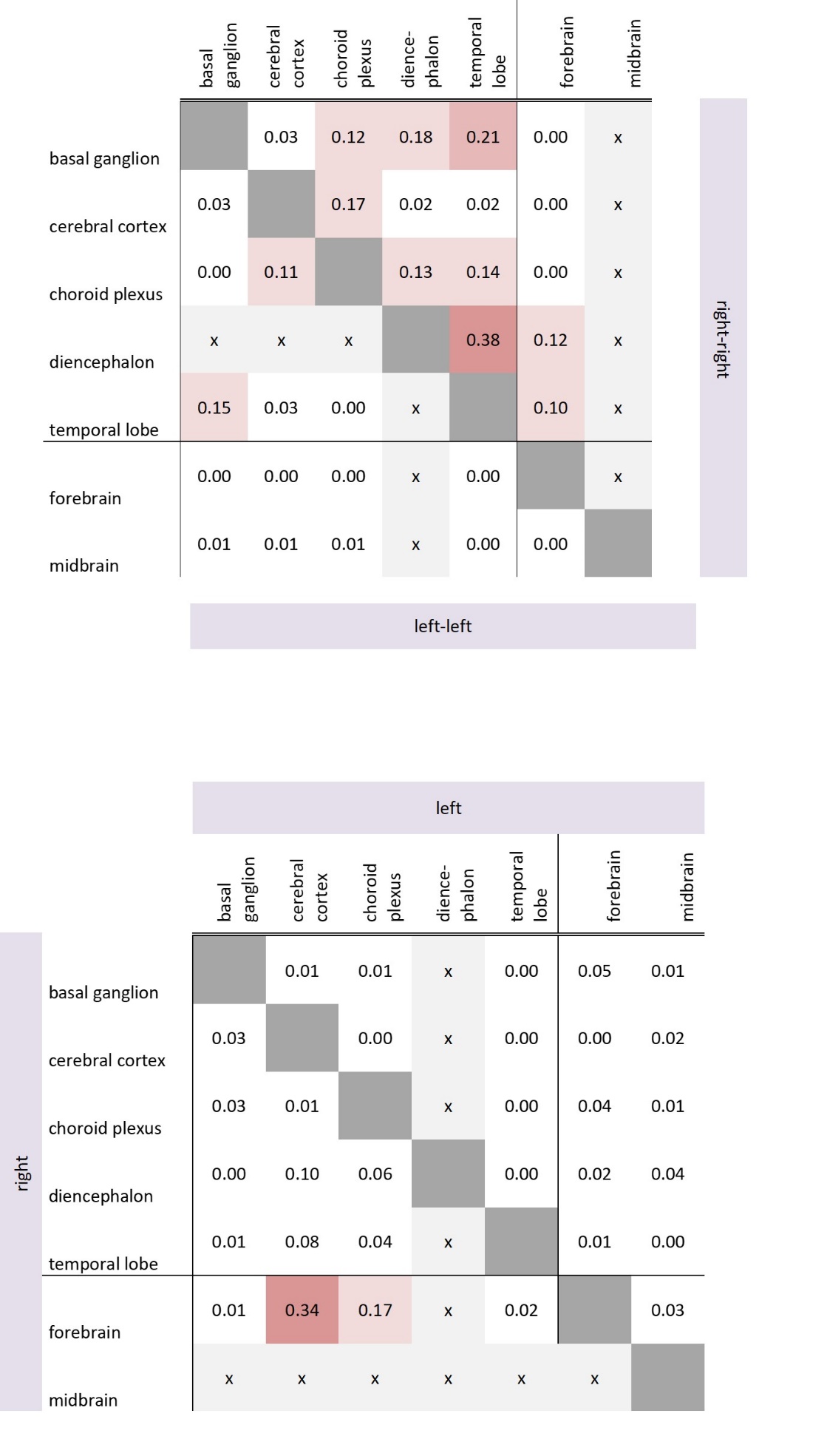


**Supp. Fig. 1.** Jaccard index showing overlap between enriched GO-terms in sides and structures. Overlap calculated for GO-terms (MSigDB v6.1) enriched with FDR<0.05 on left or right sides of structures. Top: Upper triangle shows overlap between right sides of the structures. Lower triangle shows overlap between left sides of the structures. Bottom: Overlap between opposite sides. Numbers and colours show degree of overlap. Crosses indicate lack of data. Note that the number of enriched GO-terms varies widely between structures.


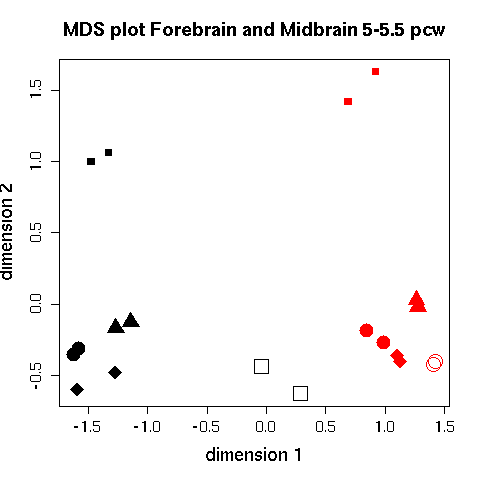


**Supp. Fig. 2**. MDS-plot based on gene expression in midbrain and forebrain samples at 5-5.5pcw. Midbrain samples are shown in blue, forebrain samples in green. Unique symbols refer to individual embryos. The smaller squares are from the female embryo.


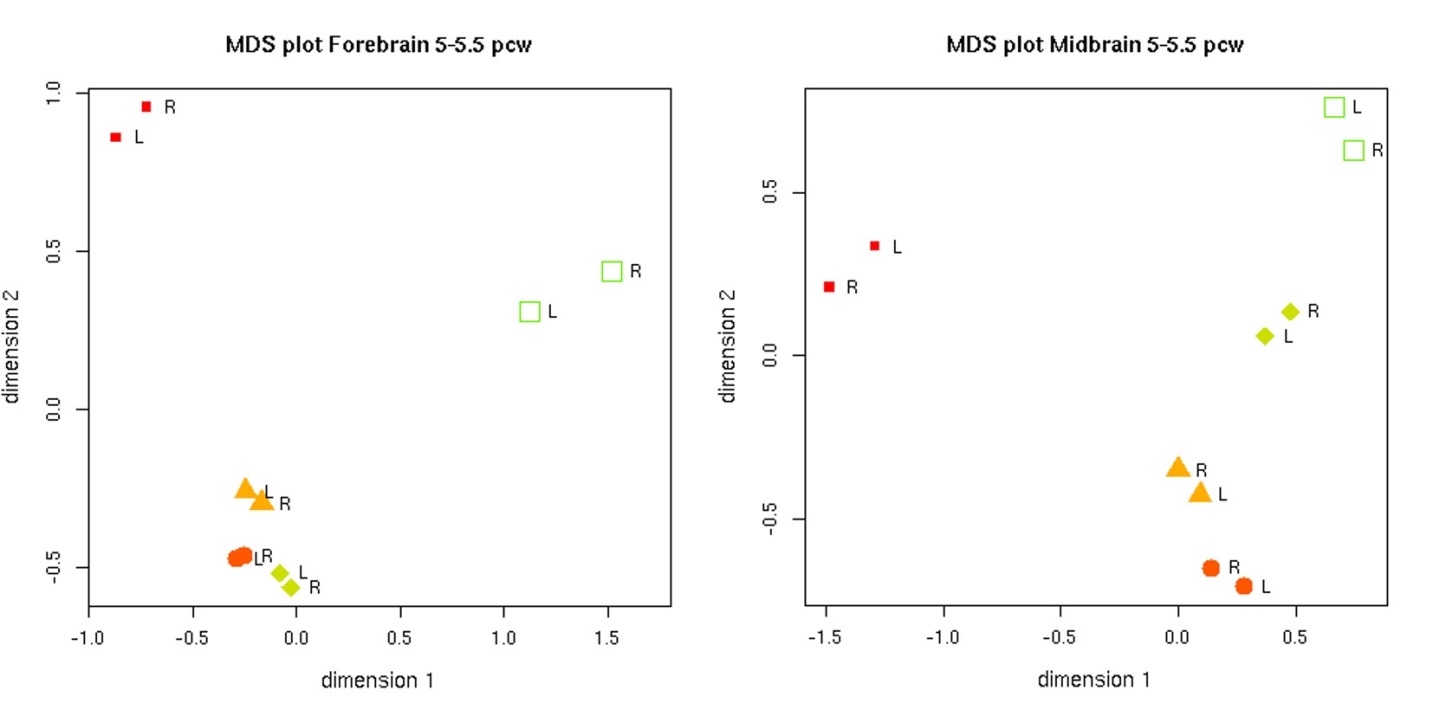


**Supp. Fig. 3**. MDS-plot based on expression data for forebrain (left) and midbrain (right) at 5-5.5 pcw. Each embryo has a different shape and colour. The smaller squares are from the female embryo. L=left. R=right.


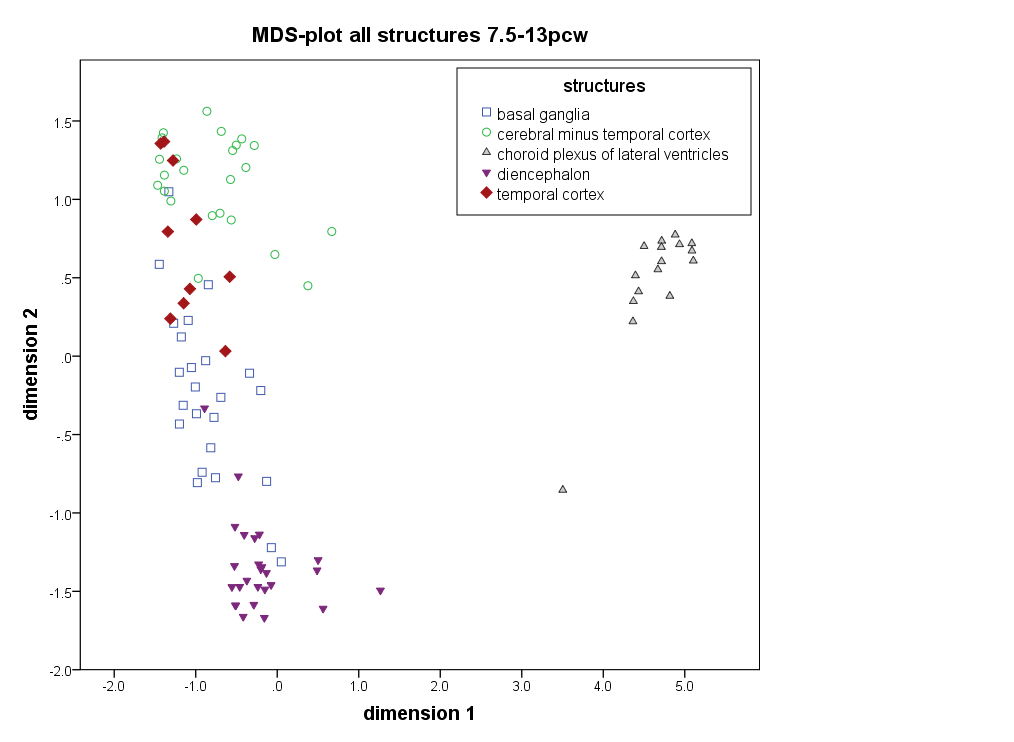


**Supp. Fig. 4**. MDS-plot based on gene expression for all samples 7.5-13 pcw, labelled by structure.


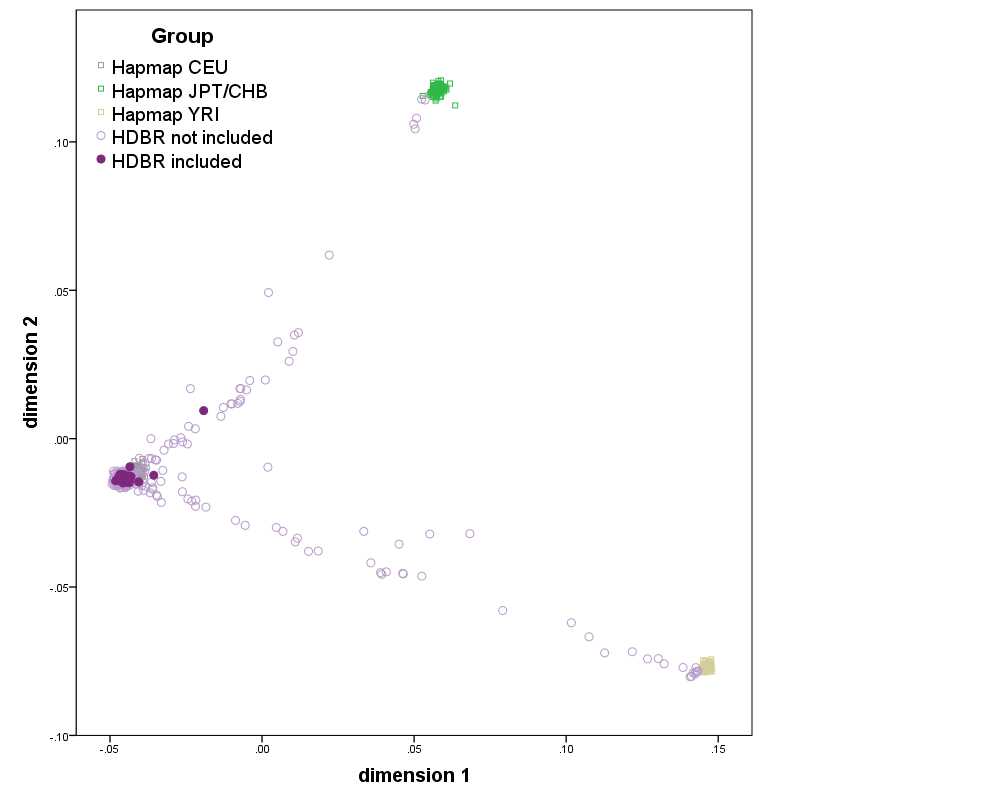


**Supp. Fig. 5**. MDS-plot based on genotypes. The plot shows the 21 foetuses 7.5-13 pcw included in our analyses (solid purple), the other embryos and foetuses made available by HDBR (open purple), and HapMap samples YRI (Yoruba in Ibadan, Nigeria), CEU (Northern and Western European ancestry) and JPT/CHB (Japanese in Tokyo, Japan/Han Chinese in Beijing, China). CEU data are hardly visible as they coincide with the bulk of the HDBR samples.


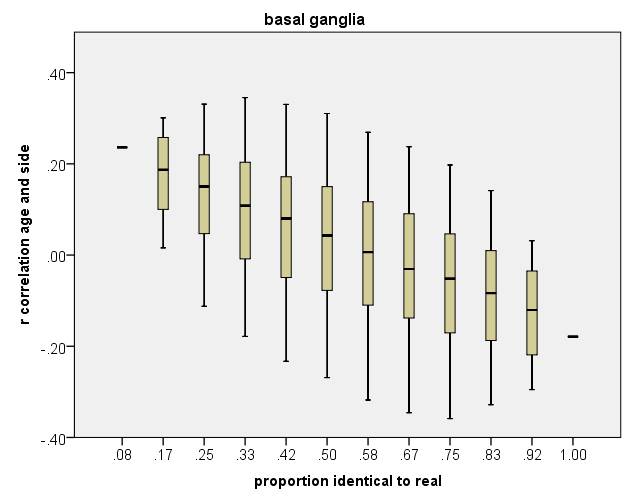

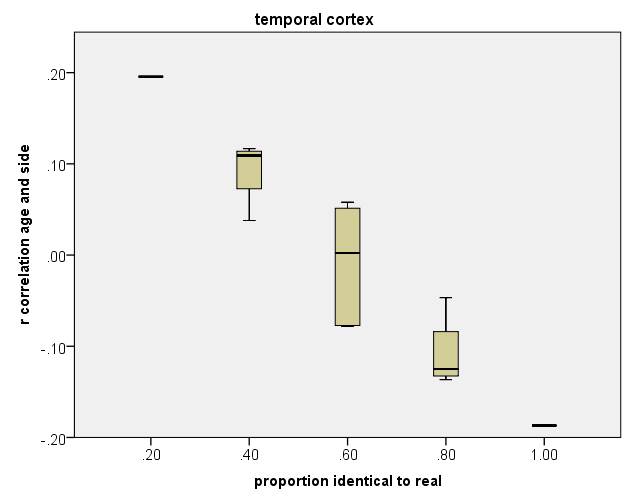

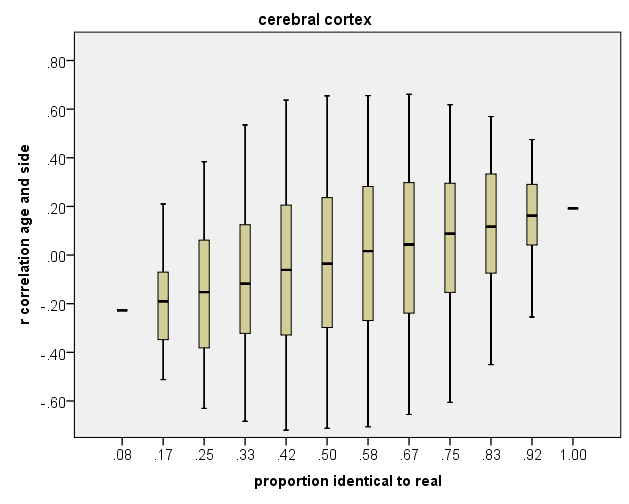

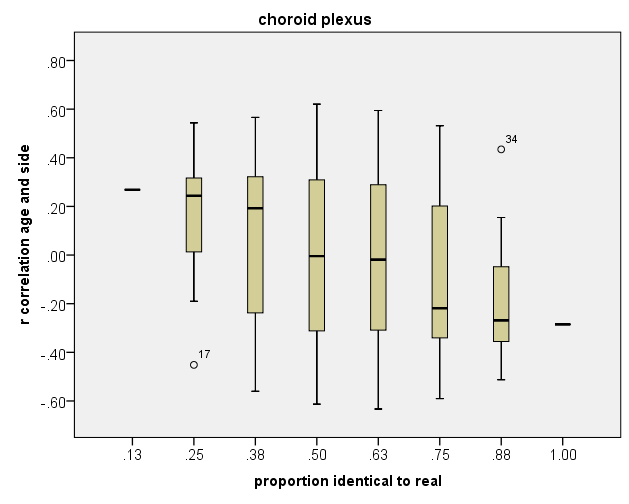

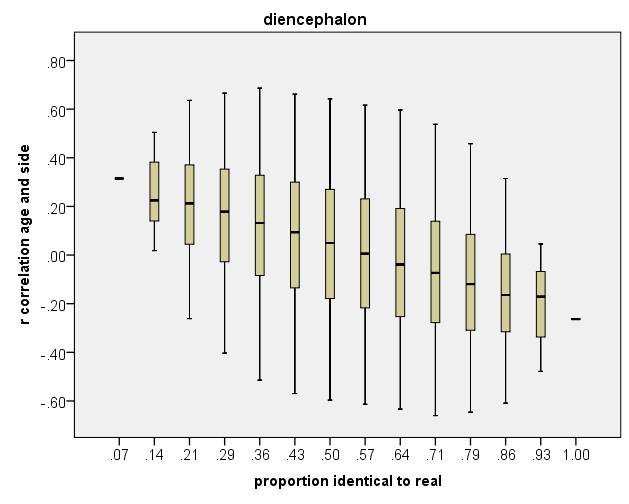


B

A

C

D

E

**Supp. Fig. 6.** For all brain structures age 7-13 pcw, box-plots are shown for the correlation between side-effects and age-effects on expression, resulting from the permutations of left and right sides (*cf* Figure 1). X-axis: proportion of foetuses in the permutations having the same left-right orientation as the real data-set. There is a clear trend for each structure whereby the average age-side correlation gets stronger, the more closely the permuted dataset matches the true dataset. Box shows the 1^st^ to 3^rd^ quartile of the data range, with the horizontal line showing the mean. Whiskers enclose 5% to 95% limits of the distribution. Outliers are plotted individually. Y-axis varies in scale. A. cerebral cortex without temporal lobe, B. temporal cortex, C. basal ganglia, D. diencephalon, E. choroid plexus of the lateral ventricles.


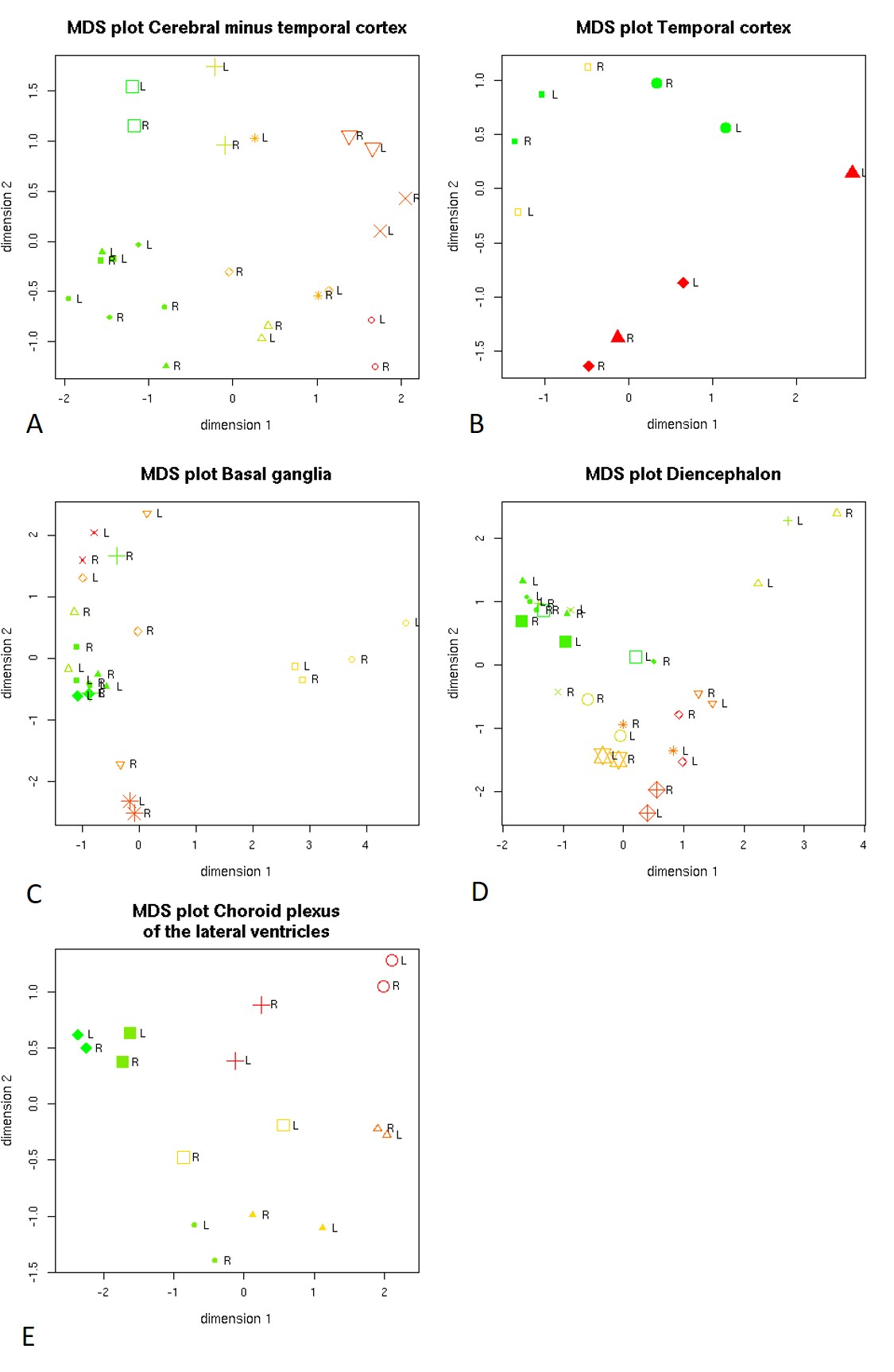


**Supp. Fig. 7**. MDS-plot based on expression data for the different brain structures, aged 7.5-13 pcw. Colours indicate age, with green the youngest, increasing through yellow and orange, to red for the oldest of the series. Identical symbols are samples from the same foetus. The smaller symbols are females. L=left. R=right. A. Cerebral cortex. The first dimension correlates with age (R=0.92, p=1.2E-10). The 2nd dimension separates sexes (p=2.4E-06). B. Temporal lobe. The second dimension shows suggestive correlation with age (R=0.69, p=0.03). None out of six dimensions clearly separates the females from the males. C. Basal ganglia. The 5th dimension (not shown) correlates with age (R=0.83, p=5.2E-07). The second dimension separates sexes (p=6.6E-05). D. Diencephalon. The second dimension correlates with age (R=0.67, p=3.7E-05). The 4th dimension (not shown) separates sexes (p=6.8E-05). E. Choroid plexus of the lateral ventricles. The first dimension shows some correlation with age (R=0.78, p=0.0004). The second dimension separates sexes (p=1.6E-07).
